# Immune-Checkpoint Expression in Breast Cancer Patients: Clinicopathological Implications: A Retrospective Case Series Study

**DOI:** 10.3390/ijms26125851

**Published:** 2025-06-18

**Authors:** Angel Quiroz-Bolaños, Antonio Quintero-Ramos, Juliana Marisol Godínez-Rubí, Ramon Franco-Topete, Porfirio Gutiérrez González, Bricia M. Gutiérrez-Zepeda, Denisse S. Becerra-Loaiza, Antonio Topete, Cesar de Loera-Rodriguez, Alicia Del Toro-Arreola, Adrián Daneri-Navarro

**Affiliations:** 1 Departamento de Fisiología, Centro Universitario de Ciencias de la Salud, Universidad de Guadalajara, 950 Sierra Mojada St., Independencia, Guadalajara 44340, Jalisco, Mexico; angelquirozqfb@gmail.com (A.Q.-B.); antonio.qramos@academicos.udg.mx (A.Q.-R.); ramon.ftopete@academicos.udg.mx (R.F.-T.); antonio.topete.camacho@usc.es (A.T.); cesar.deloera@academicos.udg.mx (C.d.L.-R.); 2 Departamento de Microbiología y Patología, Laboratorio de Patología Diagnóstica e Inmunohistoquímica (LAPADI), Centro Universitario de Ciencias de la Salud, Universidad de Guadalajara, 950 Sierra Mojada St., Independencia, Guadalajara 44340, Jalisco, Mexico; juliana.godinez@academicos.udg.mx; 3 Departamento de Matemáticas, Centro Universitario de Ciencias Exactas e Ingenierías, Universidad de Guadalajara, Guadalajara 44340, Jalisco, Mexico; porfirio.ggonzalez@academicos.udg.mx; 4 Doctorado en Genética Humana, Departamento de Biología Molecular y Genómica, Centro Universitario de Ciencias de la Salud, Universidad de Guadalajara, Guadalajara 44340, Jalisco, Mexico; 5 Departamento de Aparatos y Sistemas II, Facultad de Medicina, Decanato Ciencias de Salud, Universidad Autónoma de Guadalajara, Zapopan 45129, Jalisco, Mexico; 6 Colloids and Polymers Physics Group, Particle Physics Department, Institute of Materials (iMATUS), and Health Research Institute (IDIS), University of Santiago de Compostela, 15782 Santiago de Compostela, Spain

**Keywords:** triple-negative breast cancer, immune-checkpoint expression, overall survival

## Abstract

Immunotherapy with antibodies targeting immune checkpoints, in combination with standard therapies, is one of the areas with the most significant clinical research, particularly in aggressive tumors such as triple-negative breast cancer, where there have been relevant advances with antibodies against PD-1/PD-L1. However, it is essential to define the biological and molecular factors that influence survival and response to immunotherapy, as other immune control points, such as CTLA-4, TIM-3, LAG-3, TIGIT, and VISTA, also play a role. The immune checkpoints were studied by microarrays and immunohistochemistry in 243 samples from patients with breast cancer, according to the molecular subtype. Significant differences in PD-1, PL-1, CTLA-4, and TIGIT expression were observed between triple-negative and Her-2 tumors compared to Luminal A and Luminal B tumors. No differences in VISTA expression were observed between the different molecular subtypes. Patients with high-grade tumors showed higher PD-1, PD-L1, LAG-3, and VISTA expression than low and intermediate-grade tumors. We observed a significant difference in PD-L1/TIGIT co-expression in tumor-infiltrating cells from patients with triple-negative tumors compared to patients with Luminal A, Luminal B, and Her2+ tumors. These results are relevant in the context of clinical application.

## 1. Introduction

Breast cancer represents a serious problem globally as the leading cause of death from cancer in women [1]. The survival of patients with breast cancer is associated with molecular subtype, clinical stage, and age [2]. In particular, the triple-negative subtype (TNBC) is associated with higher recurrence, lower survival, and resistance to treatment [3]. Despite advances in surgery, chemotherapy, and different modalities based on tar-get-specific therapies, no significant benefits have been observed in patients with TNBC. The discovery and development of therapy based on immune checkpoint inhibition has revolutionized cancer treatment. However, most patients receiving this treatment (60–70%), including those with TNBC, have not shown the expected clinical benefit [4]. Treatment with antibodies against PD-1, in combination with chemotherapy, has been approved for patients with TNBC in early stages, with results of improved event-free survival and a trend to improve overall survival [5]. In patients with advanced tumors, this treatment has shown an increase in the progression-free survival (PFS) of patients with PD-L1 positive tumors, with only a response rate between 20% and 30%, without effect on overall survival [6]. For these reasons, defining the factors determining treatment response, recurrence, and resistance to treatment with immune checkpoint inhibitors against TNBC is essential. It has been described that PD-L1 expression, tumor-infiltrating lymphocytes (TILs), and tumor mutation load are predictive markers of response to treatment for some tumors [7]. Despite significant advances in our understanding of immune checkpoints, such as PD-1, PD-L1, and CTLA-4, in breast cancer, the biological and clinical significance of other immune checkpoints, including TIM-3, LAG-3, TIGIT, and VISTA, remains limited and contradictory. The objective of this study was to evaluate the expression of all these immune checkpoints in the context of their molecular profiles, clinicopathological characteristics, and patient survival.

## 2. Results

### 2.1. Patient and Tumor Characteristics

**Figure 1:**
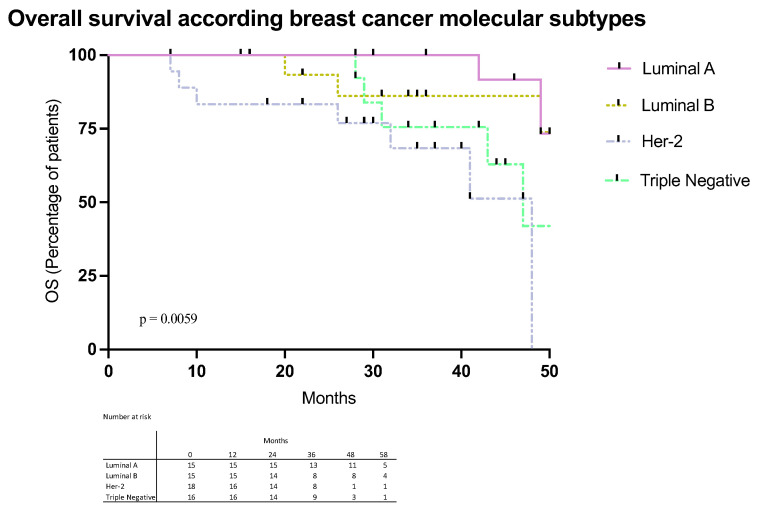
Kaplan–Meier curves of overall survival were analyzed in patients with breast cancer patients according to the molecular subtypes.

In the present study, 243 women with a diagnosis of BC (mean age 55 ± 13 years) were included. The demographic, clinical, and pathological characteristics were analyzed and shown in Table 1. No differences were observed between molecular subtypes regarding age, menopause status, and clinical stage. However, significant differences were observed between the molecular subtypes regarding tumor grade (*p* > 0.0001) and Ki67 expression (*p* > 0.0001), where a higher percentage of high grade and Ki67 was observed in patients with TN tumors. According to the molecular profile, survival analyses showed significantly lower OS (*p* = 0.0059) in patients with triple-negative and Her-2+ tumors compared to patients classified as Luminal A or Luminal B (Figure 1). We also observed significant differences according to the clinical stages, with lower OS and DFS in patients with stage IV followed by those with stage III, II, and I (*p* = 0.0001).

### 2.2. Immune Checkpoints Gene Expression

**Figure 2:**
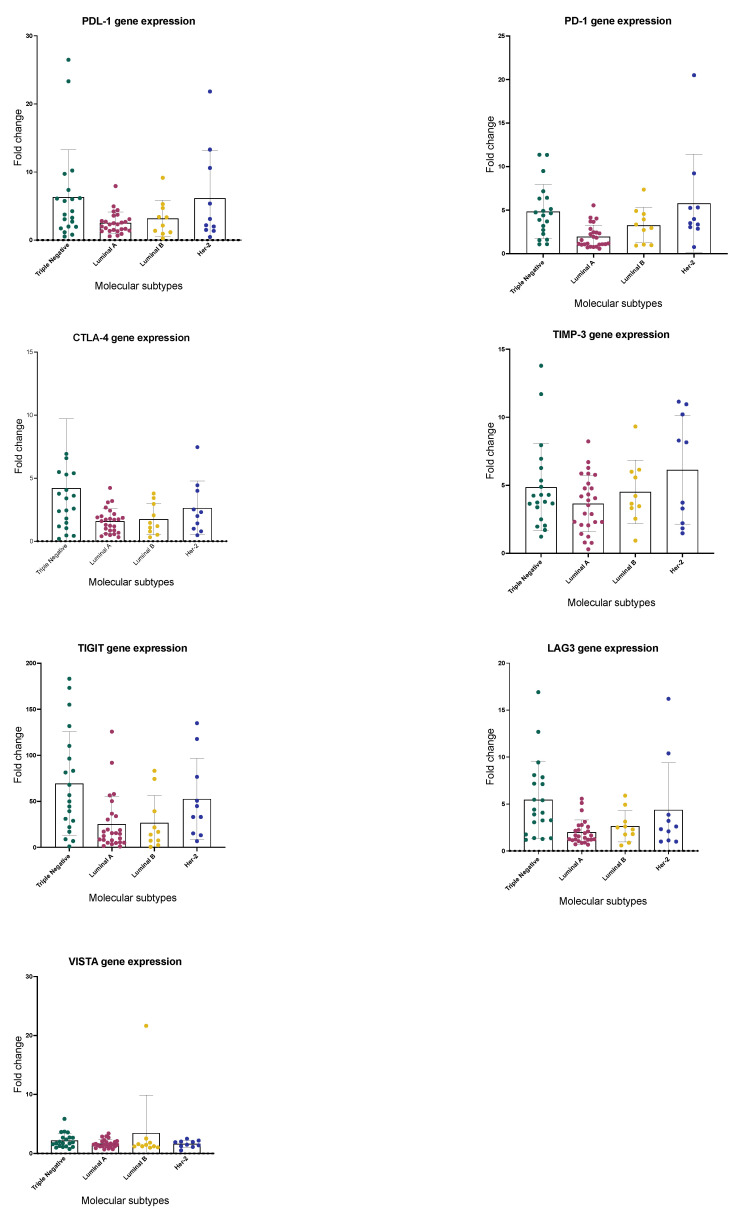
Immune checkpoints gene expression according to breast cancer molecular subtypes.

Tumors from breast cancer patients showed high heterogeneity in the expression of different immune checkpoints. TIGIT presented the highest increase with an average of 50.9-fold change (0.6–172), followed by PD-L1 4.9 (0.5–26.5), TIM-3 3.7 (0.7–10.4), LAG-3 2.9 (0.5–16.9), PD-1 2.9 (0.6–10.8), CTLA-4 2.0 (0.3–6.9) and VISTA 2.0 (0.7–5.8). The gene expression level of most immune checkpoints studied was higher in triple-negative and Her-2+ patients, particularly PD-L1, PD-1, CTLA-4, LAG-3, and TIGIT (Figure 2).

To ensure that differences in immune checkpoint expression by molecular subtype are independent of histological grade, age, and clinical stage, we evaluated the effect of these variables using the multiple regression model for all immune checkpoints. The significance remained statistically significant after adjusting for grade, age, and clinical stage in PD-1 (*p* = 0.01), TIGIT (*p* = 0.01), and LAG-3 (*p* = 0.02). However, the CTLA-4 and PDL-1 gene expression, which was statistically significant between triple-negative and Her-2 tumors versus Luminal A and Luminal B tumors in univariate analysis (*p* = 0.02 and *p* = 0.009), was no longer significant (*p* = 0.21) after adjusting for tumor grade in a multivariable regression model.

The percentage of patients with fold change gene expression > 2 was also higher in triple-negative tumors, followed by Her-2+ tumors. PD-L1 gene expression was highest in triple-negative breast cancer, where 70% of tissues showed >2-fold change, followed by Her-2+ (66%), Luminal B (52%), and Luminal A (50%). PD-1 expression was also significantly higher in tumors from triple-negative (80%) and Her-2+ (86.7%) compared to Luminal A (45%) and Luminal B (41.2%) breast tumors. CTLA-4 gene expression was also higher in triple-negative (65%) and Her-2+ (60%) tumors than gene expression in Luminal A (30%) and Luminal B (17.6%) tumors. No significant differences in TIM-3 gene expression were observed between molecular subtypes: >2-fold change in 75% of Luminal A tumors, 80% in Her-2+, 88.2% in Triple-Negative, and 88.2% in Luminal B. The most highly expressed immune checkpoint was TIGIT: >2-fold change was seen in 85% (Luminal A), 94.1% (Luminal B), 95% (triple-negative), and 100% in Her-2+ tumors. LAG-3 expression was also higher in triple-negative (75%) and Her-2+ (73.3%) tumors compared to Luminal A (45%) and Luminal B (41.18%). The immune checkpoint with the lowest expression in breast cancer was VISTA, where increased expression was observed in only 25% of Luminal A tumors, 20% in Luminal B, 6.7% in Her-2+, and 40% in the triple-negative.

Tumors with a high malignancy grade showed a higher percentage of immune checkpoints > 2: PD-L1 (87.5%), PD-1 (80.0%), CTLA-4 (68.0%), LAG-3 (79.2%), and TIM-3 (95.8%) than tumors with intermediate or low grade (Table 2). No significant differences were observed in TIGIT and VISTA compared to tumors with an intermediate and low malignancy grade. Although tumors from patients in stage IV showed a higher expression of the seven immune checkpoints studied, no significant differences were observed in tumors from patients in stages I, II, and III.

We analyzed the association of different immune checkpoints according to clinical stages and molecular profiles using Pearson’s correlation. In clinical stage I, we observed a strong correlation between PD-L1 and TIM-3 (0.83), TIGIT, and PD-L1 (0.70). A strong correlation was also observed between CTLA-4 and PD-1 with LAG-3 (0.85) in stage II. In clinical stage III, a strong correlation was also observed between PD-L1 and VISTA (0.81), PD-1 and LAG-3 (0.91), and CTLA-4 and LAG-3 (0.85). CTLA-4 and VISTA (0.90) in stage IV also showed a strong correlation. In the advanced stages, we observed a negative correlation between TIM-3 with TIGIT (−0.98), PD-1 (−0.77), and LAG-3 (−0.82). In triple-negative tumors, we observed a strong correlation between PD-1 and LAG-3 (0.87), LAG-3, and VISTA (0.77). We also observed a correlation between PD-L1 and LAG-3 (0.67) and VISTA (0.73). In Her-2+ tumors, a robust correlation was observed between PD-1 with LAG-3 (0.96) and CTLA-4 (0.85).

Overall survival and disease-free survival studies showed no significant differences between breast cancer patients with >2-fold change versus patients with <2-fold change gene expression for the seven immune checkpoints. We also did not observe significant differences in OS and DFS of patients with more than three or five immune checkpoints compared to patients with less than two immune checkpoints.

### 2.3. Expression of Immune Checkpoints by Immunohistochemistry

The immune checkpoints with the highest percentage of positive cases by immunohistochemistry were TIM-3 (74.6%) and TIGIT (71.8%), followed by VISTA (64.8%), CTLA-4 (52.1%), PD-L1 (47.9%), PD-1 (46.5%) and LAG-3 (4.2%). Triple-negative tumors were positive for LAG-3 in 9.5% of cases, while Luminal A and Her-2 were all negative cases. PD-L1 was positive in both tumor cells and in tumor-infiltrating cells, while CTLA-4, PD-1, PD-L1, TIM-3, TIGIT, and VISTA were mainly observed in tumor-infiltrating cells (Figure 2). Of the PD-L1-positive tumors, 67.6% were positive for both tumor and immune-infiltrating cells. In these cases, a significant fraction showed an intratumoral infiltrate pattern (61%) versus 39% with a peritumoral infiltrate pattern. We observed a significant difference (*p* = 0.02) in PD-L1/TIGIT co-expression in tumor-infiltrating cells from patients with triple-negative tumors (33.3%) compared to patients with Luminal A (10.5%), Luminal B (20.0%) and Her2+ (12.5%) tumors.

**Figure 3:**
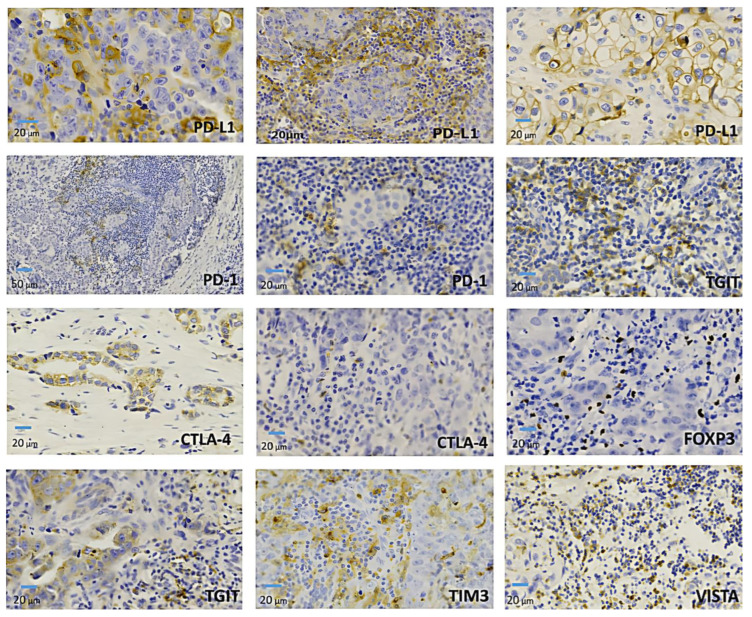
Expression of PD-L1, PD-1, CTLA-4, TIGIT, TIM-3, VISTA, and FOXP-3 of representative samples from breast cancer patients by immunohistochemistry.

In Figure 3, we observe the expression of the immune checkpoints and FOXP3 in the tumor microenvironment of representative samples from breast cancer patients. PD-L1 expression is seen on the membranes of tumor cells and tumor-infiltrating cells. PD-1 is limited to tumor-infiltrating lymphocytes, as is TGIT. The latter is also observed in the cytoplasm of some tumor cells. CTLA-4 is observed in granular deposits in the cytoplasm and membrane of infiltrating lymphocytes and tumor cells. FOXP-3 is observed in the nucleus of tumor-infiltrating lymphocytes. TIMP-3 and VISTA are observed in the cytoplasm of infiltrating lymphocytes.

Analyses assessing the concordance between molecular assays and immunohistochemistry using ROC curve analysis showed good concordance for LAG-3 (area under ROC curve 0.9888), TIGIT (area under ROC curve 0.8261), and PD-1 (area under ROC curve 0.7972) and moderate concordance for PDL-1 (0.7212) and TIMP-3 (0.7302). On the other side, the concordance for VISTA was low (0.5032).

No significant differences were observed in overall survival and overall disease-free survival between tumors positive versus those negative for all immune checkpoints. However, a tendency to a lower DFS was observed in patients positive with more than three immune checkpoints.

## 3. Discussion

In this study, it was observed that patients with triple-negative breast cancer had lower OS compared to patients with Luminal A and Luminal B tumors, associated with a higher degree of malignancy and a higher percentage of Ki67. These results agree with other studies from Latin America, reflecting the importance of having new therapeutic targets against this molecular subtype [8].

In line with new therapeutic strategies based on targets present in the tumor microenvironment, such as immune checkpoint inhibition, in this work, we focus on the study of principal immune checkpoints and their relationship with demographic, pathological, and clinical characteristics of breast cancer patients. Our results showed that the seven immune checkpoints are expressed heterogeneously but higher in samples from patients with triple-negative and Her-2+ breast cancer. Other studies have also reported this heterogeneity and increased expression of checkpoints in triple-negative and Her-2+ tumors [9,10]. In this study, we found that in addition to molecular subtypes, the expression of immune checkpoints is associated with breast cancer grade. The literature has described that increased expression of immune checkpoints is related to tumor progression, mutational load, and infiltration by immune system cells, mainly in triple-negative tumors. These facts highlight the importance of studying the role of biomarkers, including immune checkpoints, in the response and resistance to treatment with immune checkpoint inhibitors in triple-negative tumors [11]. In this work, the analyses using the multiple regression model to adjust for variables that may affect immune checkpoint expression indicate that PD-1, TIGIT, and LAG-3 are independent of histological grade, clinical stage, and age. This fact is relevant because the co-expression of these immune checkpoints has been described in tumor-infiltrating lymphocytes, indicating a state of immune exhaustion and the potential use of these biomarkers in clinical practice [12].

Our immunohistochemistry studies confirmed that PD-L1 is expressed both in tumor and infiltrating cells, while CTLA-4, PD-1, PD-L1, TIM-3, TIGIT, and VISTA were identified in infiltrating cells, both intratumorally and peritumorally. Our results are consistent with those published by other authors, who found a moderate concordance between gene expression results and immunohistochemical studies of PD-L1 and PD-1 [13]. The explanation for the better concordance between gene expression and immunohistochemistry for LAG-3, TIGIT, and PD-1 is based on the fact that these control points are expressed mainly in infiltrating T lymphocytes, which reflect a higher expression of RNA. However, differences in sensitivity and specificity between the two techniques, as well as in the characteristics of the tissues studied, cannot be ruled out.

Exhausted T cells are characterized by the progressive loss of function, proliferation, and cytokine production of CD8+ and CD4+ T cells during chronic exposure to antigens, such as in tumors and chronic infections. Exhausted T cells show increased CTLA-4, PD-1, LAG3, TIM-3, and TIGIT expression [14]. In this work, we demonstrate the significative co-expression of these immune checkpoints in tumor-infiltrating cells, suggesting a T cell exhaustion state in many of the tumors studied, mainly triple-negative and Her2+ tumors. These facts are relevant because it lays the groundwork for combining different inhibitors against immune checkpoints with greater efficacy and better safety. It was recently published, in a murine experimental model, that the joint use of antibodies against PD-L1 and LAG-3 in combination with doxorubicin has excellent potential for therapeutic application due to its efficacy and lower toxicity due to autoimmune effects [15]. The main limitations of this study are the number of patients, which hampers the power of survival studies, and the associations between immune checkpoints and clinicopathological variables.

The future expectations for our results are related to the utilization of more accurate information about the tumor microenvironment across different molecular subtypes, particularly their potential use as a biomarker, following a study with larger patient numbers. Additionally, the focus is on new checkpoints, such as TIGIT and LAG-3, as therapeutic targets.

To our knowledge, this is the first study to address the principal immune checkpoints by gene expression and immunohistochemistry in the context of molecular subtypes, clinical stages, survival, and clinicopathological variables. This contribution will be of great help in the investigation of new immune checkpoint inhibitor combinations.

## 4. Materials and Methods

### 4.1. Patients

This retrospective case series study was conducted on 273 breast cancer patients (stages I–IV) recruited for the ELLA Binational Breast Project at Instituto Jalisciense de Cancerologia, OPD-hospital Civil de Guadalajara, HE-CMNO-IMSS and HGO-CMNO-IMSS from 2006 to 2010. The study was approved by the Ethics Committee (CI-9708). Eligibility Criteria: All women of Mexican descent ages 18 years or older, diagnosis of invasive breast cancer (histologically confirmed invasive adenocarcinoma, including ductal, lobular, medullary, tubular, and mucinous cellular patterns) within the past 12 months, willing to complete a short questionnaire and consent to tissue acquisition (remaining after surgery or preoperative core needle biopsy). Exclusion Criteria: Male gender and inadequate tissue were obtained for the four main clinical markers (ER, PR, Her-2/neu, and Ki67). All patients signed the informed consent and met the inclusion and non-inclusion criteria established in the study. All samples were obtained before patients received chemotherapy and/or radiotherapy. Patient clinical and pathological information was complete, including a 5-year survival follow-up.

### 4.2. Biospecimen Collection and Immunohistochemistry Studies

Samples were collected, processed, and stored according to the procedures recommended by The Cancer Genome Atlas Program (TCGA) (https://brd.nci.nih.gov/brd/sop-compendium/show/701, accessed on 20 May 2017). Slide-mounted tissue arrays were created in a 2 × 3 configuration, with positive controls for tonsils, spleen, and lymph nodes. The following antibodies were used for immune checkpoints: Rabbit Monoclonal (CAL49) to CTLA4 ABCAM/Catalog AB237712, diluted 1/500, Rabbit Monoclonal [EPR4877(2)] to PD1 ABCAM (Cambridge, UK)/Catalog AB137132 diluted 1/250, Rabbit Monoclonal (73–10) to PD-L1 ABCAM (Cambridge, UK))/Catalog AB228415 diluted 1/500, Recombinant Anti-TIM3 Antibody (EPR22241) ABCAM (Cambridge, UK)/Catalog AB241332 diluted 1/1000, Rabbit Monoclonal (EPR20261) to LAG-3 ABCAM/Catalog AB209236 diluted 1/500, Rabbit Monoclonal [BLR047F] to TIGIT-BSA Free ABCAM (Cambridge, UK)/Catalog AB243903 diluted 1/100 and Rabbit Monoclonal [EPR21050] to VISTA ABCAM (Cambridge, UK)/Catalog AB230950 diluted 1/1000. Finally, the tissue microarrays were processed automatically using the Bond-Max Leica equipment. The negative controls were processed with the same serum used for incubating the monoclonal antibodies, and antigen recovery was performed with EDTA or citrate, as indicated in the equipment supplier’s protocols. The images were observed with a microscope (ICB Model PRISMA 209, Mexico, Mexico) at 40× magnification. They were digitized using the Aperio Scan Scope LV1 at 60× magnification and analyzed with the freely available QuPath software (https://qupath.github.io/ (accessed on 30 May 2025), Belfast, Northern Ireland) for histopathological attribute analysis using an objective and reproducible method. Scoring was performed by two experienced pathologists, who were blinded to clinical information and assessed based on the number of positive cells and staining intensity. The level of ICP expression was evaluated using the tumor cell proportion score (TPS), which represents the percentage of tumor cells with positive membrane staining for each immune checkpoint relative to the total number of tumor cells at any intensity.

### 4.3. Gene Expression

Total RNA was extracted from biopsies or surgical resections of breast cancer tissue, depending on sample availability for each patient. The pathologist confirmed adequate tumor representation in the sample used for microarray analysis. The breast tissue sample used for microarray analysis contained more than 60% tumor cells and less than 20% necrosis, as determined by hematoxylin-eosin (H&E)-stained slides. For RNA quantification and integrity, only samples with a RIN greater than or equal to 4 were used for the study. Quantification was performed using the NanoDrop kit (Wilmington, DE, USA). For normalization methods or correction, we used Agilent’s Two-Color Microarray-based Gene Expression Analysis (Santa Clara, CA, USA), which utilizes cyanine 3- and cyanine 5-labeled targets to measure gene expression in experimental and control samples. A Stratagene Universal Reference RNA (La Jolla, CA, USA) was used for the control samples. Finally, as part of data normalization, Agilent provides proprietary software (Agilent Feature Extraction, https://www.agilent.com.cn/zh-cn/product/mirna-microarray-platform/mirna-microarray-software/feature-extraction-software-228496, accessed on 30 May 2025) for image analysis and pre-processing of microarray data. RNA amplification, labeling, and hybridization (Kit), Agilent gene expression hybridization (Kit), and Agilent stabilization and drying solution (Kit) were used. A universal reference RNA was used. For the microarray chip, the following was selected: Human GE 4 × 44K v2 AMADID 026652 from Agilent (G2519F) because the 4 × 44K array was used. For the microarray design, a two-color design was used for this study. A fixed microarray design was used throughout the study; thus, the same probes were used in all samples. Agilent Feature Extraction software was used for bioinformatics, biostatistics, and data management.

### 4.4. Statistical Analysis

Descriptive statistics were used to characterize the study population. Continuous variables were reported as means ± standard deviation or medians with interquartile ranges, depending on their distribution. Categorical variables are presented as absolute frequencies and percentages. To evaluate the association between molecular subtypes of breast cancer and clinical and pathological characteristics, chi-square tests or Fisher’s exact tests were used in cases of expected low frequencies. To ensure that differences in immune checkpoint expression by molecular subtype are independent of histological grade, age, and clinical stage, we evaluated the effect of these variables using the multiple regression model for all immune checkpoints. To more accurately assess the concordance between immune checkpoint gene expression and immunohistochemistry, we performed ROC curve analyses. For the analysis of disease-free survival and overall survival, the Kaplan–Meier method was used, comparing survival curves between the different molecular subtypes using the log-rank test.

## Figures and Tables

**Table 1:** Demographic, clinical, and pathological characteristics of breast cancer patients according to hormonal and Her-2 status.

Parameter					
	H(+)Her2(−)	H(+)Her2(+)	H(−)Her2(+)	Triple-Negative	Analysis *p*
**Age**					NS
Mean (SD)	57.65 ± 12.42	56.44 ± 23.42	55.35 ± 12.76	54.00 ± 14.60	
Range	33−89	35−86	33−93	28−85	
**Menopause N (%)**					NS
Pre-menopause	37 (25)	4(22.22)	0 (27.02)	15 (34.09)	
Post-Menopause	111 (75)	14(77.78)	27 (77.98)	29 (65.91)	
**Clinical stage N (%)**					NS
I	26 (19.12)	4 (25.00)	13 (9.09)	5 (12.50)	
II	67 (49.27)	10 (62.50)	12 (36.36)	19 (47.50)	
III	37 (27.20)	1 (12.25)	13 (39.39)	15 (37.50)	
IV	6 (4.41)	1 (12.25)	5 (15.15)	1 (2.50)	
**Grade**					0.0001
Low	30 (25.42)	3 (20.00)	2 (6.06)	2 (6.45)	
Intermediate	72 (61.01)	8 (53.33)	15 (45.45)	5 (16.13)	
High	16 (13.56)	4 (26.67)	16 (48.49)	24 (77.42)	
**Ki67**					0.0001
<10%	62 (51.24)	1(5,55)	6 (20.00)	8 (21.05)	
10–19%	20 (16.53)	1 (5.55)	6 (20.00)	7 (18.42)	
≥20%	39 (32.23)	16 (88.00)	18 (60.00)	23 (60.53)	

**Table 2:** Immune checkpoints gene expression according to the breast cancer grade.

Immune Checkpoint (% Tumors > 2-Fold Change)	Low	Grade Intermediate	High
PD-L1	57.1	45.5	87.5
PD-1	57.4	51.5	80.0
CTLA-4	42.9	24.2	68.0
LAG-3	57.1	45.5	79.2
TIM-3	76.9	76.5	95.8
TIGIT	92.3	90.9	96.0
VISTA	23.1	21.2	32.0

PD-L1, PD-1, CTLA-4, LAG-3, and TIM-3 were significantly higher in high-grade tumors than in low- and intermediate-grade tumors (*p* < 0.01).

## Data Availability

The datasets presented in this article are not readily available due to confidentiality issues.

## References

[1] Sung H., Ferlay J., Siegel R.L., Laversanne M., Soerjomataram I., Jemal A., Bray F. (2021). Global cancer statistics 2020: GLOBOCAN estimates of incidence and mortality worldwide for 36 cancers in 185 countries. CA A Cancer J. Clin..

[2] De Almeida L.M., Cortes S., Vilensky M., Valenzuela O., Cortes-Sanabria L., de Souza M., Barbeito R.A., Abdelhay E., Artagaveytia N., Daneri-Navarro A. (2022). Socioeconomic, Clinical, and Molecular Features of Breast Cancer Influence Overall Survival of Latin American Women. Front. Oncol..

[3] Xiong N., Wu H., Yu Z. (2024). Advancements and challenges in triple-negative breast cancer: A comprehensive review of therapeutic and diagnostic strategies. Front. Oncol..

[4] Ma W., Xue R., Zhu Z., Farrukh H., Song W., Li T., Zheng L., Pan C.X. (2023). Increasing cure rates of solid tumors by immune checkpoint inhibitors. Exp. Hematol. Oncol..

[5] Schmid P., Cortes J., Dent R., Pusztai L., McArthur H., Kümmel S., Bergh J., Denkert C., Park Y.H., Hui R. (2022). Event-free Survival with Pembrolizumab in Early Triple-Negative Breast Cancer. N. Engl. J. Med..

[6] Zhang W., He Y., Tang Y., Dai W., Si Y., Mao F., Xu J., Yu C., Sun X. (2023). A meta-analysis of application of PD-1/PD-L1 inhibitor-based immunotherapy in unresectable locally advanced triple-negative breast cancer. Immunotherapy.

[7] Pilard C., Ancion M., Delvenne P., Jerusalem G., Hubert P., Herfs M. (2021). Cancer immunotherapy: It’s time to better predict patients’ response. Br. J. Cancer.

[8] Llera A.S., Abdelhay E.S.F.W., Artagaveytia N., Daneri-Navarro A., Müller B., Velazquez C., Alcoba E.B., Alonso I., da Quinta D.B.A., Binato R. (2022). The Transcriptomic Portrait of Locally Advanced Breast Cancer and Its Prognostic Value in a Multi-Country Cohort of Latin American Patients. Front. Oncol..

[9] Swoboda A., Nanda R. (2018). Immune Checkpoint Blockade for Breast Cancer. Cancer Treat. Res..

[10] Lan H.R., Chen M., Yao S.Y., Chen J.X., Jin K.T. (2024). Novel immunotherapies for breast cancer: Focus on 2023 findings. Int. Immunopharmacol..

[11] Emens L.A., Loi S. (2023). Immunotherapy Approaches for Breast Cancer Patients in 2023. Cold Spring Harb. Perspect. Med..

[12] Mollavelioglu B., Cetin Aktas E., Cabioglu N., Abbasov A., Onder S., Emiroglu S., Tukenmez M., Muslumanoglu M., Igci A., Deniz G. (2022). High co-expression of immune checkpoint receptors PD-1, CTLA-4, LAG-3, TIM-3, and TIGIT on tumor-infiltrating lymphocytes in early-stage breast cancer. World J. Surg. Oncol..

[13] Ren X.Y., Wu H.W., Lu J.L., Zhang Y.H., Luo Y.F., Xu Q.Q., Shen S.J., Liang Z.Y. (2018). PD1 protein expression in tumor infiltrated lymphocytes rather than PDL1 in tumor cells predicts survival in triple-negative breast cancer. Cancer Biol. Ther..

[14] Baessler A., Vignali D.A.A. (2024). T Cell Exhaustion. Annu. Rev. Immunol..

[15] Zhang C., Liu J., Gu T., Meng X., Cai X., Zhang J., Chen Y., Zhang D., Wu Y. (2025). Enhanced antitumor efficacy of bispecific antibody blocking PD-L1 and LAG-3 with doxorubicin: Mechanism and safety evaluation. Breast Cancer Res. Treat..

